# Assessing differences in contraceptive provision through telemedicine among reproductive health providers during the COVID-19 pandemic in the United States

**DOI:** 10.1186/s12978-022-01388-9

**Published:** 2022-04-22

**Authors:** Alison B. Comfort, Lavanya Rao, Suzan Goodman, Tina Raine-Bennett, Angela Barney, Biftu Mengesha, Cynthia C. Harper

**Affiliations:** 1 Department of Obstetrics, Gynecology, and Reproductive Sciences, Bixby Center for Global Reproductive Health, University of California San Francisco, 550 16th Street, 3rd floor, San Francisco, CA 94143 USA; 2 Present Address: Deloitte, Portland, OR USA; 3 Division of Research, Kaiser Permanente, Oakland, CA USA; 4 Present Address: Medicines360, San Francisco, CA USA; 5 Department of Pediatrics, University of California San Francisco, San Francisco, CA USA; 6 Department of Family and Community Medicine, Bixby Center for Global Reproductive Health, University of California San Francisco, San Francisco, CA USA

**Keywords:** Contraception, Family planning, COVID-19, Telemedicine, Service delivery, Sexual and reproductive health

## Abstract

**Background:**

Providers faced challenges in maintaining patient access to contraceptive services and public health safety during the COVID-19 pandemic. Due to increased barriers to care, providers increasingly used telemedicine for contraceptive care, curbside services, mail-order pharmacies, and on-line or home delivery of contraceptive methods, including self-administration of subcutaneous depo medroxyprogesterone acetate (DMPA-SQ). To better understand how reproductive health providers adapted service provision during the pandemic, this study assessed clinical practice changes and strategies providers adopted throughout the United States to maintain contraceptive care, particularly when clinics closed on-site, and the challenges that remained in offering contraceptive services, especially to marginalized patient populations.

**Methods:**

We surveyed U.S. providers and clinic staff (n = 907) in April 2020–January 2021, collecting data on contraceptive service delivery challenges and adaptations, including telemedicine. We assessed clinical practice changes with multivariate regression analyses using generalized linear models with a Poisson distribution and cluster robust standard errors, adjusting for clinic patient volume, practice setting, region, Title X funding, and time of survey.

**Results:**

While 80% of providers reported their clinic remained open, 20% were closed on-site. Providers said the pandemic made it more difficult to offer the full range of contraceptive methods (65%), contraceptive counseling (61%) or to meet the needs of patients in marginalized communities (50%). While only 11% of providers offered telemedicine pre-pandemic, most offered telemedicine visits (79%) during the pandemic. Some used mail-order pharmacies (35%), curbside contraceptive services (22%), and DMPA-SQ for self-administration (10%). Clinics that closed on-site were more likely to use mail-order pharmacies (aRR 1.83, 95% CI [1.37–2.44]) and prescribe self-administered DMPA-SQ (aRR 3.85, 95% CI [2.40–6.18]). Clinics closed on-site were just as likely to use telemedicine as those that remained open. Among clinics using telemedicine, those closed on-site continued facing challenges in contraceptive service provision.

**Conclusions:**

Clinics closing on-site were just as likely to offer telemedicine, but faced greater challenges in offering contraceptive counseling and the full range of contraceptive methods, and meeting the needs of marginalized communities. Maintaining in-person care for contraceptive services, in spite of staffing shortages and financial difficulties, is an important objective during and beyond the pandemic.

## Plain English Summary

Providers faced challenges in maintaining patient access to contraceptive services and public health safety during the COVID-19 pandemic. Due to increased barriers to care, providers increasingly used telemedicine for contraceptive care as well as using curbside services, mail-order pharmacies, and on-line or home delivery of contraceptive methods, including self-administration of subcutaneous depo medroxyprogesterone acetate (DMPA-SQ). This study examined use of telemedicine and other clinical practice changes among U.S. contraceptive providers during the COVID-19 pandemic. We surveyed providers (n=907) in April 2020–January 2021, collecting data on contraceptive service delivery challenges and adaptations, including telemedicine. Our analyses showed that, in response to the pandemic, many clinics adopted telemedicine to offer contraceptive services. Furthermore, clinics that were closed on-site were just as likely to start offering telemedicine visits during the pandemic as those that remained open. Despite these adaptions, clinics still faced challenges in offering the full range of contraceptive methods, providing contraceptive counseling, and meeting the needs of patients in marginalized communities. Offering telemedicine visits during the pandemic did not reduce the challenges in offering contraceptive services. Few studies have focused on providers and the clinical practice changes they rapidly achieved to maintain contraceptive access for their patients during the pandemic. Our results highlighted that telemedicine should be considered as a complement to on-site care because of the challenges in providing full services without in-person visits. Maintaining in-person care for contraceptive services, in spite of staffing shortages and financial difficulties, is an important objective during and beyond the pandemic.

## Background

Health care providers have sought to balance patient access to essential health services, including contraception, and public health safety during the COVID-19 pandemic. A national survey showed access to sexual and reproductive health care was constrained during this time; one-third of respondents reported delaying or canceling a provider visit or having trouble accessing birth control [1]. Analysis of a national sample of claims data indeed found a significant reduction in contraceptive visits across different methods during the pandemic [2]. Many aspects of the pandemic increased barriers to contraceptive services, including clinic closures, limited appointments, lack of transportation and economic hardship [1, 3–5]. Professional and public health organizations have agreed that contraception represented an essential service [6–10]. However, providers have also had to respond to social distancing mandates, changes in clinic funding, and competing demands for COVID-related care [11–13]. While some studies have focused on patient barriers to contraceptive access [4, 14], there has been limited data from health care providers on challenges in contraceptive care during the pandemic [13, 15–17], and even fewer on the equity implications.

Providers have increasingly used telemedicine [18], including for contraceptive care [16, 19–22] following the relaxation of federal privacy regulations and expansion of payment policies by the Centers for Medicare and Medicaid Services. Indeed, reimbursement for telemedicine from both public and private payers became more flexible, in addition to relaxation of certain requirements related to HIPAA-compliant communication platforms [23–25]. Some providers have also initiated other changes including curbside services, mail-order pharmacies, and on-line or home delivery of contraceptive methods, including self-administration of subcutaneous depo medroxyprogesterone acetate (DMPA-SQ) [26–28]. To understand the challenges providers have faced in meeting patient contraceptive needs and how they have adapted clinical care, we conducted a survey among clinicians and clinic staff providing contraceptive services, focusing on under-resourced clinics including community clinics, non-profits, and public health departments.

**Fig. 1:**
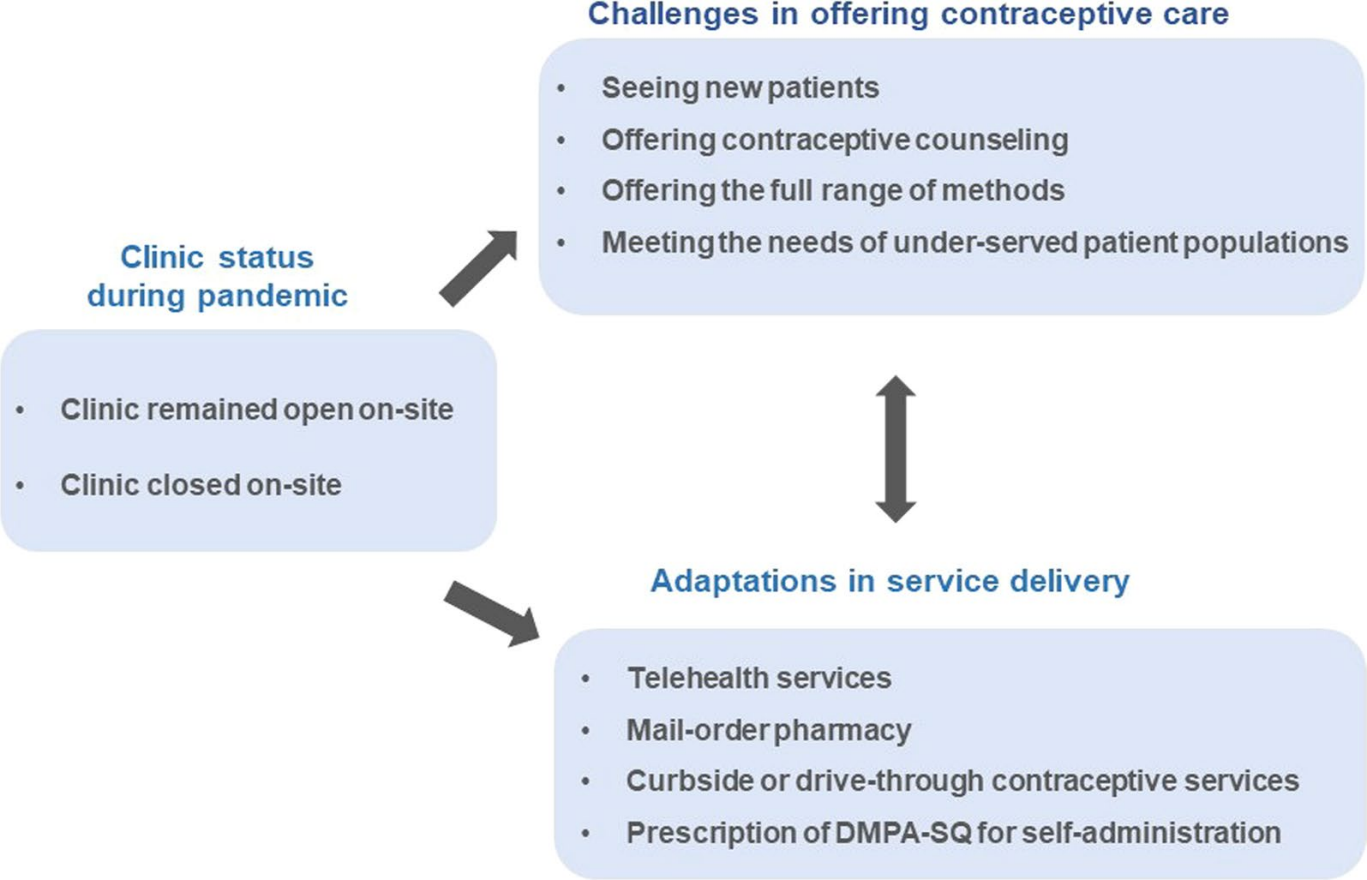
Adaptations in contraceptive service provision during the COVID-19 pandemic

The primary objective of this study was to assess clinical practice changes and the strategies providers adopted throughout the U.S. to maintain contraceptive care during the pandemic, particularly in the context of on-site clinic closures. We also sought to identify remaining challenges in offering contraceptive services, especially to marginalized patient populations (see Fig. 1 for framework for our approach). Findings from this research can help to guide clinical practice changes during and post-pandemic to ensure access to these essential services.

## Methods

### Study design and setting

We conducted a cross-sectional survey April 10, 2020 to January 29, 2021 of U.S. providers who participated in a Continuing Medical Education (CME) course sponsored by the University of California San Francisco (2015–2021). This CME course trained providers and clinic staff on contraceptive care and patient-centered counseling to increase provider capacity to offer comprehensive and high-quality contraceptive services [29]. The study sample consisted of providers and clinic staff in 43 USA states, Washington D.C., and 3 territories, covering all regions of the USA.^1^ Respondents included the full care team, with physicians, advanced practice clinicians, nurses, and staff including social workers, medical assistants, and health educators. The providers and clinic staff worked in various practice settings including primary care clinics, public health departments, family planning clinics, independent abortion care clinics, school- and college-based health centers, and hospital-based outpatient clinics.

We administered an online survey to collect data on provider type, practice setting, status of clinic at time of survey (open or closed on-site), challenges in meeting patient needs, and adaptations in contraceptive service delivery including use of telemedicine. Data also included county COVID-19 policies at time of survey (e.g. shelter-in-place order).

Eligible respondents were those providing contraceptive clinical care, counseling or education. We sent 3,497 surveys to potential study respondents, with 3–5 reminder emails. There were 907 eligible respondents, and 160 ineligible respondents (or 15% of the total) including retired, invalid email addresses, or not providing contraceptive care. Among 2,430 non-responders, we assumed a similar ineligible proportion (15%; n = 365), and removed all ineligibles (n = 525) from the response rate denominator of 3,497 yielding a denominator equal to 2972 (see American Association for the Public Opinion Research) [30]. 907 providers consented to participate and met eligibility criteria (31% response rate = 907/2972). Respondents were more likely than non-respondents to be clinicians vs. non-clinicians and in primary care. Respondents completing the survey were entered into a drawing for one of five $250 amazon gift cards.

### Study measures

#### Study outcomes: contraceptive service delivery challenges and adaptations

The first set of study outcomes assessed providers’ perspectives of pandemic-related challenges in contraceptive care. Measures included whether providers found it more difficult to: (1) see new contraceptive patients; (2) offer contraceptive counseling; (3) offer the full range of contraceptive methods; and (4) meet the needs of patients facing structural barriers to access, including housing instability, poverty, or racial/ethnic barriers. This variable was defined as 1 if respondents said they found it hard to serve patients who are experiencing homelessness, are low-income, or are BIPOC, and 0 otherwise.

For adaptations in service delivery, we measured whether providers used telemedicine or other adaptive approaches. Specifically, we asked if the clinic offered contraceptive services via: (1) telemedicine; (2) mail-order pharmacies; (3) curbside or drive-through delivery; or (4) prescription of DMPA-SQ for self-administration during the pandemic. All these variables were captured with a 4-point Likert scale (strongly agree, agree, disagree, strongly disagree)*.* We collapsed these measures into binary categories for analyses where 1 equals “strongly agree or agree” and 0 equals “strongly disagree or disagree.”

We measured variations in these challenges and adaptations as a function of whether the clinic was open on-site during the pandemic. We included covariates for providers’ practice setting, categorized as: primary care and health departments; family planning; abortion care; youth clinics, including school-based or college health centers; and hospital-based outpatient clinics. A region variable was included: Northeast, Midwest, Southeast, Southwest and West. A categorical variable for time of survey was also included (April-August 2020; September 2020–January 2021). A variable for clinic patient volume was included with large clinics defined as ≥ 800 unique patients per year [sample median]. A variable for whether the clinic received Title X funding was also included.

### Statistical analysis

We presented descriptive statistics of study measures: challenges in contraceptive services during the pandemic, adaptation of service provision, and clinic status. We also assessed differences in adaptations by practice setting with chi-squared statistics.

We conducted multivariate regression analyses of the study outcomes of challenges and adaptations in service delivery using a generalized linear model with a Poisson distribution, interpreting the estimated incidence rate ratios as relative risk ratios [31]. We used robust standard errors, clustered by clinic to account for similarities by clinic. All analyses adjusted for practice setting, region, patient volume, time when survey was administered, and Title X status. We included all observations not missing on the outcomes. An analysis comparing missingness of outcome data showed no differences between respondents by patient volume, provider type or Title X status, but there were differences by practice setting, region and time of survey. Providers at hospital settings had a higher rate of missing outcome data, compared to other practice settings (χ = 25.03, p < 0.01), as did those in the Southwest (χ = 10.57, p < 0.05). Providers who responded in the earlier pandemic period (April–August 2020) had a lower rate of missing data than those who responded later (September 2020-January 2021) (χ = 9.08, p < 0.01). For control variables, in the few cases (n = 9) where providers did not report total contraceptive patients, but did report total patient population, this value was used. For 42 missing on contraceptive patient and total patient population, we used a dummy imputation method in order not to drop these observations [32]. We used a similar approach for the 163 observations missing data for whether clinic was closed onsite during the pandemic (when used as independent variable).

Our first analysis examined the association between challenges in offering contraceptive services and whether the clinic was closed on-site during the pandemic (independent variable). Specific outcomes included challenges in seeing new patients, offering contraceptive counseling, offering the full range of contraceptive methods, and offering care to marginalized patient populations. We then assessed the use of telemedicine and other adaptations in service delivery by whether the clinic was closed on-site. Since we collected data on whether the clinic used telemedicine prior to the pandemic, we adjusted for this covariate in the analysis when use of telemedicine was the outcome. Finally, we examined whether challenges in offering contraceptive services differed by clinic status together with availability of telemedicine visits. Specifically, we interacted clinic status (open versus closed on-site) with telemedicine (offered versus not offered), with clinics open on-site and offering telemedicine as the reference group. These analyses served to better understand whether clinic closure on-site and/or availability of telemedicine were associated with reduced challenges in providing contraceptive care during the pandemic.

## Results

### Descriptive statistics of study sample

The study sample included physicians (17%), advanced practice clinicians (41%), registered nurses (16%), medical/nurse assistants (9%), health educators and social workers (11%), and clinic managers/other (7%) (Table 1). Among the physicians, 20% were obstetrician/gynecologists, 37% family practice physicians, 29% pediatricians, 5% internal medicine, and 8% other specialties. Most respondents were in non-profit and publicly funded clinics, with about one-third from clinics receiving Title X funding (and 27% “don’t know”). Respondents practiced at youth clinics/school-based health centers (SBHCs) or college health centers (36%), primary care clinics or health departments (29%), and family planning clinics (22%), with fewer from independent abortion care clinics (4%) and hospitals/other (8%). The mean patient volume was 3,184 annual contraceptive patients. Only 11% of respondents offered telemedicine prior to the pandemic.

**Table 1:** Summary characteristics of contraceptive providers (n = 907)

	n	%
Sex, n (%)		
Female	644	(95)
Male	29	(4)
Other/non-binary	8	(1)
Age (mean ± SD)	44.8 ± 11.7	
Race/ethnicity, n (%)		
White	402	(60)
Black	96	(14)
Hispanic/Latinx	100	(15)
Asian/Pacific Islander	58	(9)
Native American	13	(2)
Other	6	(1)
Provider type, n (%)		
Physician	128	(17)
Physician’s Assistant	31	(4)
Nurse Practitioner/CNM	282	(37)
Registered Nurse	120	(16)
Other Nurse/Medical Assistant	65	(9)
Health Educator/Social Worker	84	(11)
Manager/Director	19	(3)
Administrative Staff	24	(3)
Student	2	(1)
Practice setting, n (%)		
Primary care/Health department	228	(29)
Family planning	179	(22)
Youth/School-based health center/College clinic	290	(36)
Independent abortion care clinics	33	(4)
Hospital/Other	66	(8)
Clinic receives Title X funding		
Yes	228	(33)
No	268	(39)
Does not know	186	(27)
Offering telemedicine visits pre-COVID 19	76	(11)
Clinic size (from contraceptive client volume), n (%)		
Smaller clinic (volume < 800)	392	(45)
Larger clinic (volume ≥ 800)	473	(55)
Region, n (%)		
Northeast	110	(14)
Midwest	90	(11)
Southeast	206	(26)
West	200	(25)
Southwest	195	(24)
Date at survey		
April–August 2020	565	(62)
September 2020–January 2021	342	(38)

### Effects of COVID-19 pandemic on clinic service delivery

Respondents reported their clinics were affected in several ways by the COVID-19 pandemic: more than one-third shifted towards providing COVID-related care (36%) (Table 2). Forty percent said their clinic experienced shortages of medical supplies, such as personal protective equipment, medical supplies and tests. The majority (76%) reported that the caseload of contraceptive patients at their clinic decreased, 18% reported staff layoffs, and 13% reported that clinic funding decreased. Most respondents reported that their county was re-opening with restrictions (75%), and that their clinic remained open on-site during the COVID-19 pandemic (81%) (see Appendix Table 6).

**Table 2:** Challenges in meeting patients’ contraceptive needs during COVID-19 pandemic

	Overall sample (n = 907)	
	n	%
Local COVID-19 policies (at time of survey)		
Stay-at-home/shelter-in-place order	120	(15)
Re-opening with restrictions	580	(75)
Completely open	80	(10)
In what ways has your clinic been affected by the COVID-19 pandemic?		
Shift towards providing COVID-related care	259	(36)
Decreased clinic hours	315	(44)
Shortages of medical supplies/tests	288	(40)
Decreased funding to clinic	92	(13)
Increased staff layoffs	126	(18)
COVID has made it more difficult to…		
See new patients	651	(83)
Offer contraceptive counseling	473	(61)
Offer the full range of contraceptive methods	495	(65)
Meet the needs of patients who are low-income	301	(40)
Meet the needs of BIPOC patients	231	(30)
Meet the needs of patients who are experiencing homelessness	263	(35)
Meet the needs of marginalized patients^a^	381	(50)
Patient contraceptive caseload has decreased as a result of COVID-19 pandemic	536	(76)
COVID-19 pandemic has made it more challenging to offer care to patients experiencing…		
Unprotected sex	391	(51)
Forced sex	272	(36)
Intimate partner violence	345	(45)
Mental health issues	387	(51)
Patients who do not want to get pregnant	335	(44)

^a^Marginalized is a binary variable which takes on a value of 1 for patients who are homeless, low-income, or BIPOC

Challenges in meeting patients’ contraceptive needs increased. A majority of respondents (83%) reported that the pandemic made it more difficult to see new patients; offer contraceptive counseling (61%); and offer the full range of contraceptives (65%). Half of respondents reported that the pandemic made it more difficult to meet the needs of patients in marginalized communities; specifically, for patients who are low-income (40%), BIPOC (30%), or experiencing homelessness (35%). Furthermore, they cited challenges in caring for patients who did not want to become pregnant (44%), and patients experiencing unprotected sex (51%), forced sex (36%), intimate partner violence (45%), or mental health issues (51%).

### Contraceptive care via telemedicine and other service delivery adaptations

**Fig. 2:**
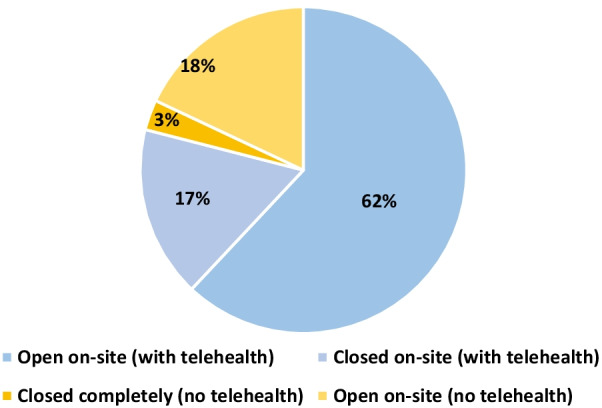
Clinic status during COVID-19 pandemic

**Fig. 3:**
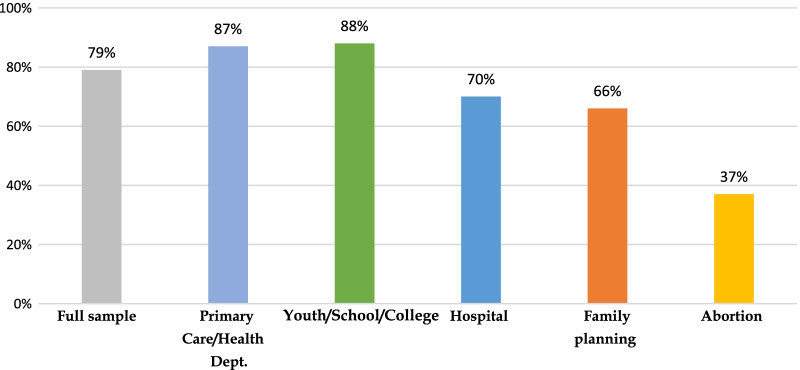
Telemedicine for contraceptive provision by practice setting during the COVID-19 pandemic

Use of telemedicine was widespread, with 79% of clinics offering telemedicine visits. Sixty-two percent of clinics were offering both onsite and telemedicine visits, while 17% were closed onsite but offering telemedicine (Fig. 2). Almost all respondents in primary care and youth clinics offered telemedicine services (87% and 88%, respectively), while two-thirds in family planning and hospital settings were offering telemedicine (66% and 70%) and about one-third in abortion care (37%) (Fig. 3). One-third of respondents (35%) said their clinic increased use of mail-order pharmacies for contraception. One-fifth (22%) were using drive-through clinics for contraceptive services. Only 45% of providers were familiar with self-administered DMPA-SQ, with 10% prescribing it.

### Challenges in meeting patients’ contraceptive needs during COVID-19 pandemic

On-site clinic closures were associated with increased challenges in meeting patients’ contraceptive needs during the pandemic in multivariate regressions, as well as with adaptations in service delivery (Tables 3, 4).

**Table 3:** Challenges in providing contraceptive services by clinic status

Independent variables	Outcomes: Clinic faced challenges in:							
	Seeing new patients		Offering contraceptive counseling		Offering the full range of methods		Offering care to marginalized patients	
	aRR	95% CI	aRR	95% CI	aRR	95% CI	aRR	95% CI
Clinic status								
Open onsite (Reference)	–	–	–	–	–	–	–	–
Closed onsite	1.15***	[1.07–1.24]	1.20**	[1.06–1.36]	1.36***	[1.20–1.54]	1.26*	[1.04–1.52]
Practice Setting								
Primary care/Health Department (Reference)		–	–	–	–	–	–	–
Family planning	0.91	[0.80–1.04]	1.06	[0.88–1.27]	1.06	[0.91–1.23]	0.87	[0.70–1.07]
Youth/School/College	1.01	[0.91–1.13]	1.16	[0.97–1.39]	1.01	[0.85–1.20]	0.94	[0.78–1.13]
Abortion	1.15**	[1.05–1.26]	1.33*	[1.01–1.75]	1.02	[0.75–1.38]	1.17	[0.83–1.67]
Hospital/other	1.00	[0.86–1.15]	1.09	[0.85–1.40]	1.18	[0.97–1.43]	0.99	[0.73–1.34]
Region								
Northeast (Reference)	–	–	–	–	–	–	–	–
Midwest	0.99	[0.91–1.09]	0.85	[0.69–1.05]	0.87	[0.70–1.09]	1.04	[0.79–1.37]
Southeast	0.92	[0.83–1.02]	0.82*	[0.69–0.97]	0.93	[0.79–1.10]	0.83	[0.64–1.07]
West	0.90*	[0.83–0.99]	0.88	[0.74–1.04]	0.78**	[0.66–0.93]	1.08	[0.86–1.37]
Southwest	0.91	[0.83–1.00]	0.97	[0.82–1.14]	0.76*	[0.61–0.94]	1.01	[0.78–1.32]
Time of survey								
April–August 2020 (Reference)	–	–	–	–	–	–	–	–
Sept 2020–Jan 2021	1.01	[0.93–1.09]	1.05	[0.91–1.21]	0.97	[0.85–1.12]	1.04	[0.86–1.27]
Clinic funding								
Not title X or does not know (Reference)	–	–	–	–	–	–	–	–
Title X	0.95	[0.87–1.04]	1.05	[0.92–1.19]	0.97	[0.84–1.11]	1.02	[0.87–1.21]
Patient volume								
Smaller clinic (Reference)	–	–	–	–	–	–	–	–
Larger clinic	0.98	[0.92–1.05]	0.80***	[0.71–0.91]	0.89*	[0.80–1.00]	0.95	[0.81–1.11]
Observations	653		651		634		650	

Standard errors are clustered by clinic. Coefficients for dummy variables representing missing data are not displayed

*aRR* adjusted relative risk ratio, *CI* confidence interval

*p ≤ 0.05, **p ≤ 0.01, ***p ≤ 0.001

**Table 4:** Adaptations in service delivery by clinic status

Independent variables	Outcomes:							
	Telemedicine		Curbside contraceptive services		Mail-order		Prescribing DMPA-SQ	
	aRR	95% CI	aRR	95% CI	aRR	95% CI	aRR	95% CI
Clinic status								
Open onsite (reference)	–	–	–	–	–	–	–	–
Closed onsite	1.12	[0.98–1.28]	0.63*	[0.39–1.00]	1.83***	[1.37–2.44]	3.85***	[2.40–6.18]
Practice setting								
Primary care/Health Department. (Reference)	–	–	–	–	–	–	–	–
Family planning	0.77***	[0.67–0.87]	3.02***	[1.99–4.58]	0.96	[0.74–1.25]	1.49	[0.97–2.29]
Youth/School/College	0.99	[0.90–1.08]	2.01**	[1.28–3.15]	0.79	[0.59–1.07]	0.85	[0.47–1.52]
Abortion	0.50*	[0.26–0.93]	0.38	[0.05–3.07]	0.56	[0.24–1.29]	4.46**	[1.61–12.31]
Hospital/Other	0.82*	[0.68–0.98]	0.83	[0.38–1.84]	0.82	[0.50–1.36]	1.01	[0.29–3.50]
Region								
Northeast (Reference)	–	–	–	–	–	–	–	–
Midwest	1.04	[0.91–1.19]	3.45**	[1.41–8.45]	0.82	[0.52–1.30]	1.39	[0.41–4.69]
Southeast	0.80*	[0.67–0.96]	2.67*	[1.14–6.24]	0.72	[0.50–1.03]	2.16	[0.83–5.66]
West	1.05	[0.93–1.18]	3.19**	[1.36–7.52]	1.03	[0.74–1.45]	1.08	[0.34–3.41]
Southwest	0.95	[0.82–1.09]	2.27	[0.95–5.44]	0.63*	[0.42–0.95]	1.12	[0.38–3.31]
Time of survey								
April–August 2020 (Reference)	–	–	–	–	–	–	–	–
Sept 2020– Jan 2021	0.97	[0.89–1.06]	1.03	[0.76–1.39]	1.27	[0.94–1.70]	1.37	[0.79–2.38]
Clinic funding								
Not title X or DK (Reference)	–	–	–	–	–	–	–	–
Title X	1.05	[0.96–1.16]	1.88***	[1.38–2.55]	1.24	[0.98–1.58]	2.53***	[1.55–4.11]
Patient volume								
Smaller clinic (Reference)	–	–	–	–	–	–	–	–
Larger clinic	1.10*	[1.00–1.21]	1.10	[0.83–1.45]	1.16	[0.93–1.45]	1.22	[0.82–1.82]
Telemedicine pre-pandemic								
Offered at clinic	1.24***	[1.16–1.32]						
Observations	649		648		570		649	

Standard errors are clustered by clinic. Coefficients for dummy variables representing missing data are not displayed

*aRR* adjusted relative risk ratio, *CI* confidence interval

*p ≤ 0.05, **p ≤ 0.01, ***p ≤ 0.001

Among clinics that remained open on-site during the pandemic, 81% were offering oral contraceptives/patch/vaginal ring on-site, 68% insertion and removal for implants and 65% for IUDs. Clinics that were closed on-site not surprisingly were more likely to face difficulty seeing new patients (adjusted relative risks [aRR] 1.15, 95% CI [1.07–1.24]) (Table 3), offering contraceptive counseling (aRR 1.20, 95% CI [1.06–1.36]), or offering the full range of contraceptive methods (aRR 1.36, 95% CI [1.12–1.45]). Clinics that were closed on-site were also more likely to face difficulty in offering care to marginalized patients (aRR 1.26, 95% CI [1.04–1.52]).

### Adaptation of service delivery and clinic closure on-site

Clinics that were closed on-site were significantly more likely use mail-order pharmacies (aRR 1.83, 95% CI 1.37–2.44]), and prescribe self-administered DMPA-SQ (aRR 3.85, 95% CI [2.40–6.18]) (Table 4). Clinics that closed on-site were less likely to offer curbside contraceptive services (aRR 0.63, 95% CI [0.39–1.00]). There were no detectable differences by clinic closure on-site and use of telemedicine during the pandemic when we adjusted for use of telemedicine pre-pandemic; indeed, clinics that offered telemedicine pre-pandemic were more likely to offer it during the pandemic.

Of note, there were differences in adaptations by practice setting, with primary care clinics more likely to offer telemedicine compared to family planning, abortion care, and hospital settings (p < 0.05). Compared to FP clinics, youth clinics were more likely to offer telemedicine visits (p < 0.01). Clinics receiving Title X funding were more likely to offer curbside contraceptive services and prescribe DMPA-SQ.

The final set of analyses compared clinic status (open on-site or closed) and availability of telemedicine visits. The results showed that, even among clinics offering telemedicine, those that closed on-site compared to those that remained open on-site (reference group) faced more challenges with contraceptive care (Table 5). Among clinics offering telemedicine visits, those that closed on-site faced more challenges than those staying open onsite in seeing new patients (aRR 1.14, 95% CI [1.05–1.23]), offering contraceptive counseling (aRR 1.19, 95% CI [1.03–1.36]), offering the full range of methods (aRR 1.37, 95% CI [1.20–1.57]) and serving patients from marginalized groups (aRR 1.23, 95% CI [1.01–1.49]). Using tests of equality of coefficients, we see that among those that stayed open, offering telemedicine did not significantly reduce challenges in offering contraceptive services. Similarly, among those that were closed on-site, offering telemedicine was not associated with lower challenges in offering contraceptive counseling. However, among clinics that were closed on-site, those that offered telemedicine were more likely to face challenges in offering the full range of methods compared to those that were not offering telemedicine (χ^2^ = 4.79, p < 0.05).

**Table 5:** Challenges in providing contraceptive services by clinic status

Independent variables	Outcomes: clinic faced challenges in:							
	Seeing new patients		Offering contraceptive counseling		Offering the full range of methods		Offering care to marginalized patients	
	aRR	95% CI	aRR	95% CI	aRR	95% CI	aRR	95% CI
Clinic status								
Open onsite + telemedicine (Reference)		–	–	–	–	–	–	–
Open onsite + no telemedicine	0.96	[0.85–1.09]	1.02	[0.85–1.23]	1.00	[0.83–1.22]	0.82	[0.64–1.04]
Closed onsite + telemedicine	1.14**	[1.05–1.23]	1.19*	[1.03–1.36]	1.37***	[1.20–1.57]	1.23*	[1.01–1.49]
Closed onsite + no telemedicine	1.08	[0.95–1.23]	1.06	[0.80–1.40]	0.88	[0.63–1.24]	1.04	[0.59–1.86]
Practice setting								
Primary care/Health Department (Reference)		–	–	–	–	–	–	–
Family planning	0.92	[0.80–1.05]	1.05	[0.87–1.27]	1.06	[0.90–1.24]	0.90	[0.73–1.12]
Youth/School/College	1.01	[0.90–1.13]	1.16	[0.97–1.39]	1.00	[0.84–1.19]	0.95	[0.79–1.13]
Abortion	1.17**	[1.05–1.30]	1.32	[0.99–1.76]	1.01	[0.74–1.39]	1.28	[0.87–1.89]
Hospital/Other	0.99	[0.85–1.14]	1.06	[0.82–1.36]	1.15	[0.94–1.40]	1.02	[0.76–1.37]
Region								
Northeast (Reference)	–	–	–	–	–	–	–	–
Midwest	1.00	[0.91–1.10]	0.86	[0.69–1.06]	0.87	[0.69–1.08]	1.04	[0.79–1.36]
Southeast	0.92	[0.83–1.02]	0.82*	[0.69–0.97]	0.92	[0.78–1.09]	0.86	[0.66–1.11]
West	0.90*	[0.83–0.99]	0.88	[0.74–1.05]	0.78**	[0.66–0.93]	1.08	[0.86–1.36]
Southwest	0.91	[0.83–1.00]	0.96	[0.81–1.13]	0.75*	[0.60–0.93]	1.03	[0.79–1.33]
Time of survey								
April–August 2020 (Reference)	–	–	–	–	–	–	–	–
Sept 2020–Jan 2021	1.00	[0.93–1.09]	1.04	[0.90–1.19]	0.98	[0.85–1.12]	1.05	[0.86–1.27]
Clinic funding								
Not title X or DK (Reference)	–	–	–	–	–	–	–	–
Title X	0.96	[0.88–1.04]	1.06	[0.93–1.20]	0.98	[0.85–1.12]	1.02	[0.86–1.20]
Patient volume								
Smaller clinic (Reference)	–	–	–	–	–	–	–	–
Larger clinic	0.98	[0.91–1.05]	0.80***	[0.70–0.91]	0.89*	[0.80–1.00]	0.93	[0.80–1.09]
Observations	649		647		630		646	

Standard errors are clustered by clinic. Coefficients for dummy variables representing missing data are not displayed

*aRR* adjusted relative risk ratio, *CI* confidence interval

*p ≤ 0.05, **p ≤ 0.01, ***p ≤ 0.001

## Discussion

This study investigated how the COVID-19 pandemic affected contraceptive provision, and findings showed that providers adapted service delivery in several ways, most importantly via telemedicine. There was a substantial increase in use of telemedicine, from 11% offering it pre-pandemic to 79% during the pandemic. On a smaller scale, providers increased use of mail-order pharmacies, curbside contraceptive services, and prescription of DMPA-SQ for self-administration. Providers and staff in clinics that closed on-site were just as likely as those that remained open on-site to offer telemedicine visits, but they were more likely to adapt service delivery in other ways, such as use of mail-order pharmacies and prescribing DMPA-SQ, to address patient contraceptive needs. Despite using telemedicine, providers continued to experience challenges in offering contraceptive care, and especially in meeting the needs of underserved communities. Results highlight that even among those that closed on-site, adopting telemedicine did not significantly reduce challenges in contraceptive service provision. Closing on-site services meant that providers continued to find it difficult during the COVID-19 pandemic to see new patients, offer contraceptive counseling, and offer the full the range of contraceptive methods. Furthermore, these results showed providers were concerned about being able to sufficiently address related health needs including mental health and intimate partner violence, which increased during the pandemic [33–35].

It is critically important to consider who is unable to use telemedicine. Concerns have been raised about the digital divide, along socio-economic, racial/ethnic, and geographic lines [5, 36]. These structural disparities, already exacerbated in the pandemic, have to be considered in access to contraception [37–39]. Early pandemic evidence points to increased barriers to sexual and reproductive health services among low-income and BIPOC populations [3]. Our study found that providers reported significantly more difficulty offering care for marginalized patient populations at the clinics that closed on-site. Many patients, including those living crowded housing, may lack internet connection or privacy for telemedicine [20]. It is also important to avoid assumptions about how patients desire to access care, which may be at the clinic itself. Further research is necessary, using an equity lens, to better understand patients’ perspective on accessing care through telemedicine.

Results showed telemedicine was rapidly adopted across practice settings during the pandemic and has many potential patient benefits. A recent study of providers (n = 172), with advanced family planning training, showed that almost all started using telemedicine for contraceptive services during the pandemic and thought it should be expanded afterwards [21]. Another recent study found a significant increase in use of telemedicine for contraception initiation and continuation among practices not previously using this modality pre-pandemic [16]. Feasibility of telemedicine for contraceptive counseling and provision of oral contraceptives has been reported [20]. Other research noted the use of digital modes, such as text messaging [22]. While not all providers were able to offer telemedicine, these findings indicate a large role for telemedicine in access to contraceptive services.

Our results highlighted that telemedicine should be considered as a complement to on-site care because of the challenges in providing full services without in-person visits. Primary care clinics are a good example, as almost three-quarters remained open, while also providing telemedicine visits. In contrast, many of the youth-serving clinics closed on-site. As schools and colleges open up, it is also essential to re-open health centers for in-person services. There may be financial and organizational pressures to transition towards telemedicine as a substitute to on-site clinics; it is important to consider that a patient-centered care approach will require a combination of in-person visits alongside telemedicine [21].

Advocacy will be needed to ensure sustainable reimbursement for telemedicine, as for other new services adapted in the pandemic, after special provisions are no longer in place [27]. DMPA-SQ for self-administration, recently included in CDC’s updated Selected Practice recommendations [40], will need to be included on formularies. One pandemic study in California showed that one-third of intramuscular depo medroxyprogesterone acetate (DMPA-IM) users were interested in a switch to DMPA-SQ to avoid clinic visits [28]. Our results of low provider familiarity reveal a need for provider training [28].

Going forward, there is a need to explore further the health equity implications of telemedicine for contraceptive services, and to understand how other new service delivery modes can help to augment contraceptive access outside of clinical hours.

The main study strengths included the timeliness of the data in showing adaptations to contraceptive care during the COVID-19 pandemic. While this sample may not be generalizable to all U.S. providers, it included individuals from 43 states, Washington DC, and 3 USA territories, across diverse practice settings and healthcare provider roles. The study has certain limitations: the analyses cannot distinguish directionality between challenges in meeting patients’ contraceptive needs and service delivery adaptations, but reveals a significant association between both and shows the ingenuity of providers at under-resourced clinics during this difficult time. The data did not capture what percent of contraceptive visits were conducted via telemedicine versus in-person and future research should examine these trends. There were differences in response rates by practice setting, region and timing of survey. Those in hospital settings, from the Southwest, and those who completed the survey later in the pandemic had lower response rates. Additionally, the effects of the pandemic varied across region and through time: we included control variables for region and date of survey, but it was not feasible to use an interaction term due to sample size restrictions. Finally, the response rate from this survey was low, though it was similar to other recent studies [15].

## Conclusions

While some research has looked at patient barriers to reproductive health services during the pandemic [4], few have focused on providers and the clinical practice changes they rapidly achieved to maintain contraceptive access for their patients during the pandemic. Findings from this study likely reveal greater adaptations than have yet occurred across the country, as these providers had sought continuing education in contraceptive care; however, they point to the very real possibility of more widespread changes we may expect to see in the future, with the continued use of telemedicine. While telemedicine and these other approaches hold promise, comprehensive contraceptive care requires in-person visits as well. There may well be financial pressures to reduce in-person visits now that telemedicine has become widely available. However, access to clinics can be important for hard-to-reach patients and to address important related sensitive health needs.

## Data Availability

The datasets used and/or analyzed during the current study are available from the corresponding author on reasonable request.

## Appendix

See Table 6.

**Table 6:** Challenges in meetings patients’ contraceptive needs during COVID-19 pandemic by time of survey (n = 908)

	April–May 2020 (n = 223)		June–July 2020 (n = 341)		August 2020–January 2021 (n = 343)		Overall sample (n = 908)	
	n	%	n	%	n	%	n	%
Local COVID-19 policies (at time of survey)								
Stay-at-home/shelter-in-place order	61	(29)	19	(7)	40	(14)	120	(15)
Re-opening with restrictions	146	(70)	244	(85)	190	(67)	580	(75)
Completely open	3	(1)	25	(9)	50	(18)	78	(10)
In what ways has your clinic been affected by the COVID-19 pandemic?								
Shift towards providing COVID-related care	88	(41)	84	(32)	87	(37)	259	(36)
Decreased clinic hours	108	(50)	112	(43)	95	(40)	315	(44)
Shortages of medical supplies/tests	96	(45)	99	(38)	93	(39)	288	(40)
Decreased funding to clinic	27	(13)	35	(14)	30	(13)	92	(13)
Increased staff layoffs	31	(14)	54	(21)	41	(17)	126	(18)
COVID has made it more difficult to…								
See new patients	185	(83)	232	(81)	234	(84)	651	(83)
Offer contraceptive counseling	140	(63)	159	(57)	174	(63)	473	(61)
Offer the full range of contraceptive methods	160	(72)	174	(63)	161	(63)	495	(65)
Meet the needs of patients who are low-income	88	(42)	109	(39)	104	(39)	301	(40)
Meet the needs of BIPOC patients	62	(30)	82	(29)	87	(32)	231	(30)
Meet the needs of patients who are experiencing homelessness	80	(38)	92	(33)	91	(34)	263	(35)
Meet the needs of patients in marginalized communities^a^	116	(55)	133	(48)	132	(49)	381	(50)
Patient contraceptive caseload has decreased as a result of COVID-19 pandemic	168	(78)	203	(78)	165	(70)	536	(76)
COVID-19 pandemic has made it more challenging to offer care to patients experiencing…								
Unprotected sex	121	(56)	140	(50)	130	(48)	391	(51)
Forced sex	83	(40)	105	(38)	84	(31)	272	(36)
Intimate partner violence	100	(47)	133	(48)	112	(42)	345	(45)
Mental health issues	107	(50)	138	(49)	142	(53)	387	(51)
Patients who do not want to get pregnant	95	(45)	126	(45)	113	(42)	334	(44)

^a^Patients in marginalized communities is a binary variables which takes on a value of 1 for patients who are homeless, low-income, or BIPOC

## Abbreviations

aRR: Adjusted risk ratio
CME: Continuing Medical Education
DMPA-SQ: Subcutaneous depo medroxyprogesterone acetate
SBHC: School-based health centers

## Footnotes

1 USA states included: Alaska, Arizona, Arkansas, California, Colorado, Connecticut, Delaware, Florida, Georgia, Hawaii, Idaho, Illinois, Indiana, Kansas, Kentucky, Louisiana, Maine, Maryland, Massachusetts, Michigan, Minnesota, Mississippi, Missouri, Montana, Nebraska, New Jersey, New Mexico, New York, North Carolina, Ohio, Oklahoma, Oregon, Pennsylvania, Rhode Island, South Carolina, Tennessee, Texas, Utah, Virginia, Washington, West Virginia, Wisconsin, Wyoming. US territories included Guam, Northern Mariana Islands and Puerto Rico.
